# 2-Formyl-3-hydr­oxy-9,10-anthroquinone

**DOI:** 10.1107/S1600536808032224

**Published:** 2008-10-22

**Authors:** Nor Hadiani Ismail, Che Puteh Osman, Khalijah Awang, Sri Nurestri Abdul Malek, Seik Weng Ng

**Affiliations:** a Faculty of Applied Sciences, Universiti Teknologi MARA, 40450 Shah Alam, Malaysia; b Department of Chemistry, University of Malaya, 50603 Kuala Lumpur, Malaysia; c Institute of Biological Sciences, University of Malaya, 50603 Kuala Lumpur, Malaysia

## Abstract

The mol­ecule of the title compound, C_15_H_8_O_4_, is approximately planar. An intra­molecular O—H⋯O hydrogen bond is observed between the hydr­oxy and formyl groups. The crystal used was a nonmerohedral twin, with a minor twin component of 15.9%.

## Related literature

For anti­leshmanial and anti­plasmodial activities, see: Sittie *et al.* (1999 ▶). For the treatment of twinned diffraction data, see: Spek (2003 ▶).
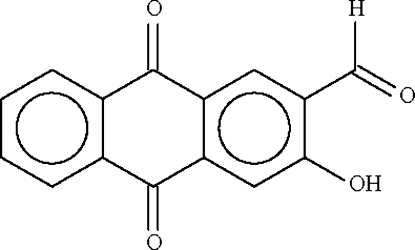


## Experimental

### 

#### Crystal data

C_15_H_8_O_4_
*M* *_r_* = 252.21
Triclinic, 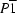

*a* = 6.9194 (2) Å
*b* = 8.0650 (2) Å
*c* = 10.7601 (3) Å
α = 86.250 (2)°
β = 83.214 (2)°
γ = 64.692 (2)°
*V* = 538.96 (3) Å^3^
*Z* = 2
Mo *K*α radiation
μ = 0.11 mm^−1^
*T* = 100 (2) K
0.22 × 0.04 × 0.04 mm

#### Data collection

Bruker SMART APEXII area-detector diffractometer
Absorption correction: none
4946 measured reflections
2419 independent reflections
1880 reflections with *I* > 2σ(*I*)
*R*_int_ = 0.024

#### Refinement

*R*[*F* ^2^ > 2σ(*F* ^2^)] = 0.087
*wR*(*F* ^2^) = 0.343
*S* = 1.11
2419 reflections
173 parameters
H-atom parameters constrained
Δρ_max_ = 0.49 e Å^−3^
Δρ_min_ = −0.44 e Å^−3^

### 

Data collection: *APEX2* (Bruker, 2007 ▶); cell refinement: *SAINT* (Bruker, 2007 ▶); data reduction: *SAINT*; program(s) used to solve structure: *SHELXS97* (Sheldrick, 2008 ▶); program(s) used to refine structure: *SHELXL97* (Sheldrick, 2008 ▶); molecular graphics: *X-SEED* (Barbour, 2001 ▶); software used to prepare material for publication: *publCIF* (Westrip, 2008 ▶).

## Supplementary Material

Crystal structure: contains datablocks I, global. DOI: 10.1107/S1600536808032224/ci2681sup1.cif

Structure factors: contains datablocks I. DOI: 10.1107/S1600536808032224/ci2681Isup2.hkl

Additional supplementary materials:  crystallographic information; 3D view; checkCIF report

## Figures and Tables

**Table 1:** Hydrogen-bond geometry (Å, °)

*D*—H⋯*A*	*D*—H	H⋯*A*	*D*⋯*A*	*D*—H⋯*A*
O2—H2⋯O1	0.84	2.00	2.635 (5)	132

**Fig. 1.**
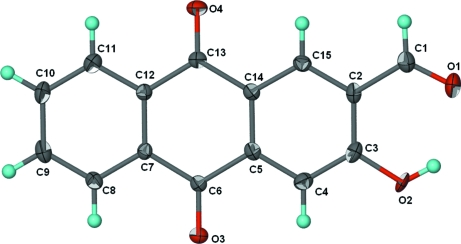
The molecular structure of the title compound, showing 70% probability displacement ellipsoids. H atoms are drawn as spheres of arbitrary radii.

## supplementary crystallographic information

### Experimental

*Rennellia elliptica* Korth from the Rubiaceae family was collected from Kuala Keniam, Pahang, Malaysia. The root was chopped into small pieces and dried. The dried sample (1 kg) was ground and then extracted successively with hexane, dichloromethane and methanol. The dichloromethane extract was concentrated *in vacuo* to give 27 g crude extract. The crude extract was fractionated by column chromatography. The column (60 cm *X* 5 cm) was packed with acid-washed silica gel and eluted with hexane, dichloromethane and methanol. Nine fractions were obtained, and 3-hydroxy-2-formyl-9,10-anthraquinone (41.5 mg) was isolated from the third fraction (hexane:dichloromethane, 30:70) by slow evaporation of the solvent mixture. The yellow crystals obtained were washed with acetone.

### Refinement

Carbon- and oxygen-bound H-atoms were placed in calculated positions (C—H = 0.95 Å and O—H = 0.84 Å) and were included in the refinement in the riding model approximation, with *U*(H) set to 1.2–1.5*U*_eq_(C,*O*). The crystal studied was a non-merohedral twin. The *TwinRotMat* in *PLATON* (Spek, 2003) gave the twin law as (-1 0 0, 0 - 1 0, -0.343 - 0.049 1), whose inclusion in the refinement lowered the *R* index from 11.3 to 8.7%. The twin component refined to 18.9%. The refinement is deemed satisfactory although the *wR*_2_ value for all reflections is somewhat high. The structure has a long C5–C14 bond; as the anisotropic displacement parameters are normal, the likely reason is localization of the double bonds in the ring. On the other hand, the C13–C14 bond is somewhat short.

### Crystal data

**Table:**

C_15_H_8_O_4_	*Z* = 2
*M**_r_* = 252.21	*F*(000) = 260
Triclinic, *P*1	*D*_x_ = 1.554 Mg m^−^^3^
Hall symbol: -P 1	Mo *K*α radiation, λ = 0.71073 Å
*a* = 6.9194 (2) Å	Cell parameters from 1888 reflections
*b* = 8.0650 (2) Å	θ = 3.3–28.3°
*c* = 10.7601 (3) Å	µ = 0.11 mm^−^^1^
α = 86.250 (2)°	*T* = 100 K
β = 83.214 (2)°	Block, yellow
γ = 64.692 (2)°	0.22 × 0.04 × 0.04 mm
*V* = 538.96 (3) Å^3^	

### Data collection

**Table:**

Bruker SMART APEXII area-detector diffractometer	1880 reflections with *I* > 2σ(*I*)
Radiation source: fine-focus sealed tube	*R*_int_ = 0.024
graphite	θ_max_ = 27.5°, θ_min_ = 2.8°
ω scans	*h* = −8→8
4946 measured reflections	*k* = −10→10
2419 independent reflections	*l* = −13→13

### Refinement

**Table:**

Refinement on *F*^2^	Primary atom site location: structure-invariant direct methods
Least-squares matrix: full	Secondary atom site location: difference Fourier map
*R*[*F*^2^ > 2σ(*F*^2^)] = 0.087	Hydrogen site location: inferred from neighbouring sites
*wR*(*F*^2^) = 0.343	H-atom parameters constrained
*S* = 1.11	*w* = 1/[σ^2^(*F*_o_^2^) + (0.1778*P*)^2^ + 2.2381*P*] where *P* = (*F*_o_^2^ + 2*F*_c_^2^)/3
2419 reflections	(Δ/σ)_max_ = 0.001
173 parameters	Δρ_max_ = 0.49 e Å^−^^3^
0 restraints	Δρ_min_ = −0.44 e Å^−^^3^

### Fractional atomic coordinates and isotropic or equivalent isotropic displacement parameters (Å^2^)

**Table:**

	*x*	*y*	*z*	*U*_iso_*/*U*_eq_	
O1	0.3155 (6)	0.6486 (5)	0.0888 (3)	0.0251 (8)	
O2	0.2501 (6)	0.3493 (5)	0.0941 (3)	0.0242 (8)	
H2	0.2611	0.4345	0.0490	0.029*	
O3	0.2366 (5)	−0.0898 (4)	0.4383 (3)	0.0153 (7)	
O4	0.2779 (5)	0.4648 (4)	0.6688 (3)	0.0172 (7)	
C1	0.3060 (7)	0.6395 (6)	0.2041 (4)	0.0178 (9)	
H1	0.3171	0.7336	0.2474	0.021*	
C2	0.2786 (7)	0.4901 (6)	0.2772 (4)	0.0143 (9)	
C3	0.2544 (7)	0.3500 (6)	0.2187 (4)	0.0161 (9)	
C4	0.2356 (7)	0.2059 (6)	0.2894 (4)	0.0152 (9)	
H4	0.2187	0.1116	0.2500	0.018*	
C5	0.2419 (6)	0.2016 (5)	0.4183 (4)	0.0123 (8)	
C6	0.2335 (6)	0.0406 (5)	0.4916 (4)	0.0112 (8)	
C7	0.2285 (6)	0.0417 (5)	0.6299 (4)	0.0112 (8)	
C8	0.2117 (7)	−0.1043 (6)	0.7003 (4)	0.0139 (8)	
H8	0.1986	−0.1999	0.6601	0.017*	
C9	0.2144 (7)	−0.1091 (6)	0.8286 (4)	0.0179 (9)	
H9	0.2031	−0.2083	0.8766	0.021*	
C10	0.2336 (7)	0.0310 (6)	0.8883 (4)	0.0188 (9)	
H10	0.2353	0.0269	0.9767	0.023*	
C11	0.2503 (7)	0.1762 (6)	0.8187 (4)	0.0173 (9)	
H11	0.2645	0.2709	0.8594	0.021*	
C12	0.2462 (6)	0.1835 (5)	0.6895 (4)	0.0113 (8)	
C13	0.2641 (6)	0.3403 (6)	0.6165 (4)	0.0126 (8)	
C14	0.2621 (6)	0.3426 (5)	0.4787 (4)	0.0120 (8)	
C15	0.2815 (7)	0.4851 (6)	0.4072 (4)	0.0133 (8)	
H15	0.2968	0.5801	0.4468	0.016*	

### Atomic displacement parameters (Å^2^)

**Table:**

	*U*^11^	*U*^22^	*U*^33^	*U*^12^	*U*^13^	*U*^23^
O1	0.038 (2)	0.0258 (18)	0.0125 (16)	−0.0155 (16)	−0.0033 (13)	0.0054 (13)
O2	0.046 (2)	0.0283 (18)	0.0063 (15)	−0.0226 (17)	−0.0062 (13)	0.0020 (12)
O3	0.0194 (15)	0.0131 (14)	0.0147 (15)	−0.0079 (12)	−0.0028 (11)	−0.0009 (11)
O4	0.0228 (16)	0.0148 (15)	0.0165 (15)	−0.0103 (13)	−0.0003 (12)	−0.0039 (11)
C1	0.021 (2)	0.017 (2)	0.016 (2)	−0.0078 (17)	−0.0040 (16)	0.0035 (16)
C2	0.0146 (19)	0.0149 (19)	0.0119 (19)	−0.0053 (16)	−0.0011 (14)	0.0030 (15)
C3	0.017 (2)	0.021 (2)	0.0103 (19)	−0.0080 (17)	−0.0030 (15)	0.0010 (15)
C4	0.016 (2)	0.016 (2)	0.014 (2)	−0.0074 (16)	−0.0007 (15)	−0.0025 (15)
C5	0.0106 (18)	0.0107 (18)	0.0135 (19)	−0.0032 (14)	0.0010 (14)	0.0003 (14)
C6	0.0099 (17)	0.0105 (18)	0.0121 (19)	−0.0036 (14)	−0.0001 (14)	−0.0004 (14)
C7	0.0109 (18)	0.0114 (18)	0.0103 (18)	−0.0038 (14)	−0.0025 (13)	0.0011 (14)
C8	0.0145 (19)	0.0118 (18)	0.015 (2)	−0.0053 (15)	−0.0023 (15)	0.0013 (14)
C9	0.019 (2)	0.017 (2)	0.017 (2)	−0.0081 (17)	−0.0012 (16)	0.0055 (16)
C10	0.024 (2)	0.022 (2)	0.0110 (19)	−0.0100 (18)	−0.0009 (16)	0.0026 (16)
C11	0.021 (2)	0.018 (2)	0.013 (2)	−0.0084 (17)	−0.0008 (16)	−0.0009 (15)
C12	0.0108 (18)	0.0112 (18)	0.0109 (18)	−0.0039 (14)	−0.0002 (14)	−0.0005 (14)
C13	0.0112 (18)	0.0125 (19)	0.0139 (19)	−0.0046 (15)	−0.0015 (14)	−0.0010 (14)
C14	0.0116 (18)	0.0112 (18)	0.0127 (19)	−0.0044 (14)	0.0001 (14)	−0.0002 (14)
C15	0.0138 (19)	0.0112 (18)	0.014 (2)	−0.0049 (15)	0.0006 (14)	−0.0017 (14)

### Geometric parameters (Å, °)

**Table:**

O1—C1	1.234 (5)	C7—C8	1.398 (5)
O2—C3	1.345 (5)	C7—C12	1.403 (5)
O2—H2	0.84	C8—C9	1.380 (6)
O3—C6	1.222 (5)	C8—H8	0.95
O4—C13	1.226 (5)	C9—C10	1.397 (6)
C1—C2	1.464 (6)	C9—H9	0.95
C1—H1	0.95	C10—C11	1.388 (6)
C2—C15	1.400 (6)	C10—H10	0.95
C2—C3	1.407 (6)	C11—C12	1.391 (6)
C3—C4	1.391 (6)	C11—H11	0.95
C4—C5	1.390 (6)	C12—C13	1.487 (5)
C4—H4	0.95	C13—C14	1.483 (6)
C5—C14	1.410 (6)	C14—C15	1.387 (6)
C5—C6	1.494 (5)	C15—H15	0.95
C6—C7	1.485 (5)		
			
C3—O2—H2	120.0	C9—C8—H8	120.1
O1—C1—C2	122.8 (4)	C7—C8—H8	120.1
O1—C1—H1	118.6	C8—C9—C10	120.4 (4)
C2—C1—H1	118.6	C8—C9—H9	119.8
C15—C2—C3	119.7 (4)	C10—C9—H9	119.8
C15—C2—C1	119.1 (4)	C11—C10—C9	120.0 (4)
C3—C2—C1	121.2 (4)	C11—C10—H10	120.0
O2—C3—C4	117.9 (4)	C9—C10—H10	120.0
O2—C3—C2	121.8 (4)	C10—C11—C12	120.2 (4)
C4—C3—C2	120.3 (4)	C10—C11—H11	119.9
C5—C4—C3	119.2 (4)	C12—C11—H11	119.9
C5—C4—H4	120.4	C11—C12—C7	119.6 (4)
C3—C4—H4	120.4	C11—C12—C13	119.4 (4)
C4—C5—C14	121.2 (4)	C7—C12—C13	121.1 (3)
C4—C5—C6	118.4 (4)	O4—C13—C14	121.0 (4)
C14—C5—C6	120.3 (4)	O4—C13—C12	121.0 (4)
O3—C6—C7	121.3 (4)	C14—C13—C12	118.0 (3)
O3—C6—C5	120.5 (4)	C15—C14—C5	119.0 (4)
C7—C6—C5	118.1 (3)	C15—C14—C13	119.7 (4)
C8—C7—C12	120.1 (4)	C5—C14—C13	121.3 (4)
C8—C7—C6	118.9 (4)	C14—C15—C2	120.5 (4)
C12—C7—C6	121.0 (3)	C14—C15—H15	119.8
C9—C8—C7	119.7 (4)	C2—C15—H15	119.8
			
O1—C1—C2—C15	176.5 (4)	C10—C11—C12—C7	−1.0 (6)
O1—C1—C2—C3	−2.1 (7)	C10—C11—C12—C13	180.0 (4)
C15—C2—C3—O2	179.8 (4)	C8—C7—C12—C11	1.0 (6)
C1—C2—C3—O2	−1.7 (7)	C6—C7—C12—C11	−177.2 (4)
C15—C2—C3—C4	−0.7 (7)	C8—C7—C12—C13	−180.0 (3)
C1—C2—C3—C4	177.9 (4)	C6—C7—C12—C13	1.8 (6)
O2—C3—C4—C5	179.3 (4)	C11—C12—C13—O4	−1.5 (6)
C2—C3—C4—C5	−0.3 (7)	C7—C12—C13—O4	179.4 (4)
C3—C4—C5—C14	1.4 (6)	C11—C12—C13—C14	179.2 (4)
C3—C4—C5—C6	−176.7 (4)	C7—C12—C13—C14	0.2 (6)
C4—C5—C6—O3	5.1 (6)	C4—C5—C14—C15	−1.6 (6)
C14—C5—C6—O3	−173.0 (4)	C6—C5—C14—C15	176.4 (3)
C4—C5—C6—C7	−176.8 (4)	C4—C5—C14—C13	178.7 (4)
C14—C5—C6—C7	5.1 (6)	C6—C5—C14—C13	−3.2 (6)
O3—C6—C7—C8	−4.6 (6)	O4—C13—C14—C15	1.6 (6)
C5—C6—C7—C8	177.4 (4)	C12—C13—C14—C15	−179.1 (4)
O3—C6—C7—C12	173.7 (4)	O4—C13—C14—C5	−178.7 (4)
C5—C6—C7—C12	−4.4 (6)	C12—C13—C14—C5	0.6 (6)
C12—C7—C8—C9	−0.5 (6)	C5—C14—C15—C2	0.7 (6)
C6—C7—C8—C9	177.7 (4)	C13—C14—C15—C2	−179.6 (4)
C7—C8—C9—C10	0.0 (7)	C3—C2—C15—C14	0.4 (6)
C8—C9—C10—C11	0.0 (7)	C1—C2—C15—C14	−178.1 (4)
C9—C10—C11—C12	0.5 (7)		

### Hydrogen-bond geometry (Å, °)

**Table:**

*D*—H···*A*	*D*—H	H···*A*	*D*···*A*	*D*—H···*A*
O2—H2···O1	0.84	2.00	2.635 (5)	132

## References

[bb1] Barbour, L. J. (2001). *J. Supramol. Chem.* **1**, 189–191.

[bb2] Bruker (2007). *APEX2* and *SAINT* Bruker AXS Inc., Madison, Wisconsin, USA.

[bb3] Sheldrick, G. M. (2008). *Acta Cryst.* A**64**, 112–122. 10.1107/S0108767307043930 18156677

[bb4] Sittie, A. A., Lemnmich, E., Olsen, C. E., Hviid, L., Kharazmi, A., Nkrumah, F. K. & Christensen, S. B. (1999). *Planta Med.* **65**, 259–261. 10.1055/s-2006-960473 10232074

[bb5] Spek, A. L. (2003). *J. Appl. Cryst.* **36**, 7–13.

[bb6] Westrip, S. P. (2008). *publCIF* In preparation.

